# Supplementary material for: Design of a self-driven probiotic-CRISPR/Cas9 nanosystem for sono-immunometabolic cancer therapy
Source: Nat Commun. 2022 Dec 22;13:7903. doi: 10.1038/s41467-022-35580-z (PMC9780327; doi:10.1038/s41467-022-35580-z)
Supplement: Supplementary file 1 — Supplementary Information [file 41467_2022_35580_MOESM1_ESM.pdf]

Supplementary Information for

Design of a self-driven probiotic-CRISPR/Cas9 nanosystem for sono-immunometabolic cancer therapy

Jifeng Yu[1#], Bangguo Zhou[2, 3, 4#], Shen Zhang [2, 3, 4#], Haohao Yin[1, 2, 3, 4*], Liping Sun[2, 3, 4], Yinying Pu[2, 3, 4], Boyang Zhou[1], Yikang Sun[1], Xiaolong Li[1], Yan Fang[2, 3, 4], Lifan Wang[1], Chongke Zhao[1], Dou Du[2, 3, 4], Yan Zhang[2, 3, 4] & Huixiong Xu [1*]

[#]These authors contributed equally.

*Corresponding author email: xu.huixiong@zs-hospital.sh.cn; yin.haohao@zs-hospital.sh.cn.

Supplementary Figures

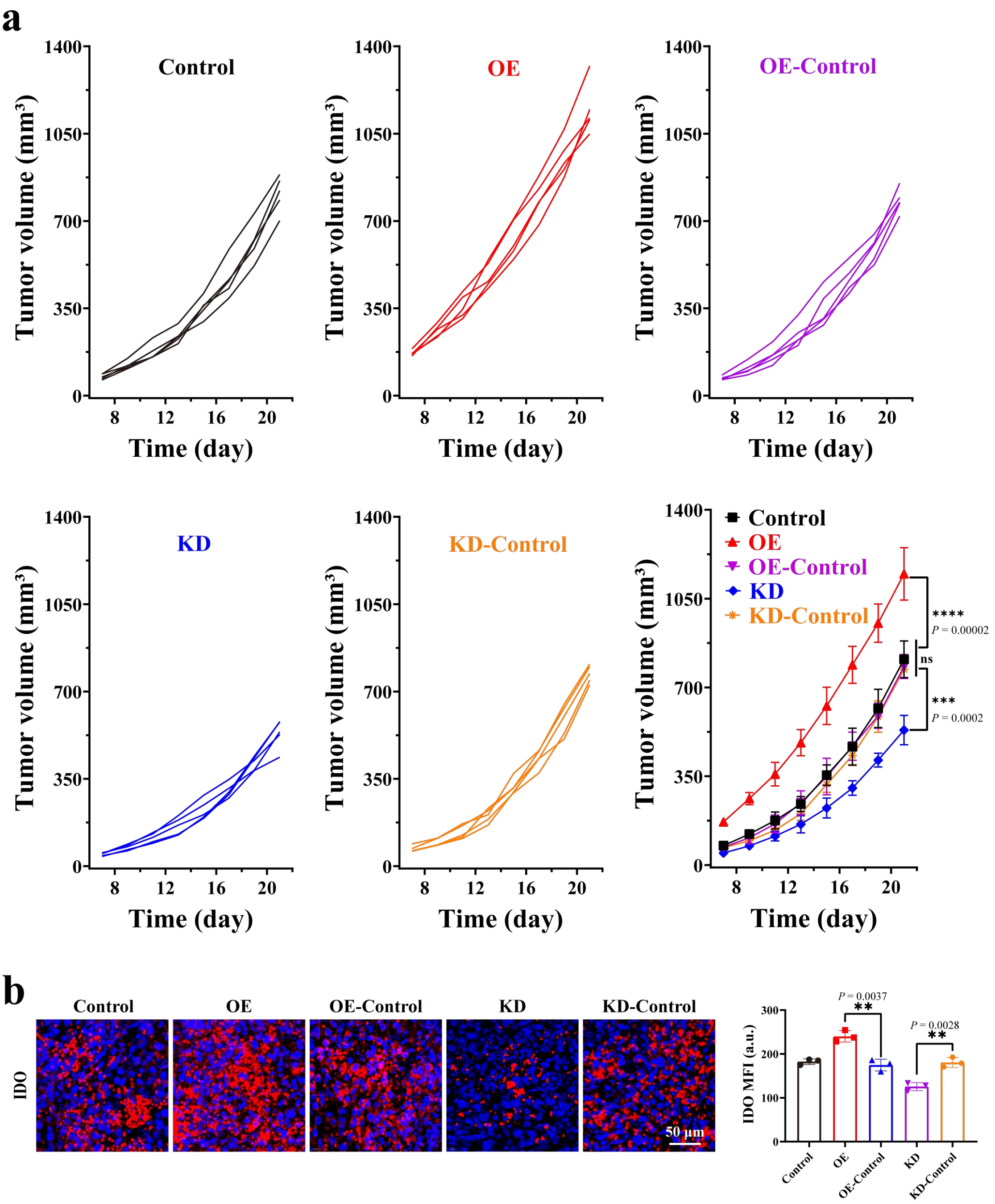

Supplementary Figure 1. 1×10^6 4T1 cells transfected with different plasmids (including Control (without treating), OE (*IDO1* over expression plasmid), OE-Control (Untreated plasmid for OE), KD (*IDO1* knock down plasmid), KD-Control (Untreated plasmid for KD)) were injected into the second left breast pad of 6-week-old female Balb/c mice at day 0. Tumor volume of mice were measured every 2 days during days 7-21. The mice in each group were euthanized after 21 days of injection, and the tumor tissues were collected for immunofluorescence staining (IDO). (a) Separate and integrated tumor growth curves (n = 5 mice per group, statistical differences were calculated using two-way ANOVA with the Geisser-Greehouse correction, match values are stacked into a subcolumn, data were expressed as means ± SD in integrated tumor growth curves, *P < 0.05, **P

< 0.01, ***$P < 0.001$, ****$P < 0.0001$.) and (b) Images of IDO immunofluorescence staining and corresponding mean fluorescence intensity of 4T1 tumor-bearing mouse after different treated. DAPI was used to stain the nucleus of the cell (blue), and the IDO was stained with anti-IDO antibodies (red) (n = 3 biologically independent samples, statistical differences were calculated using two-tailed unpaired Student's t-test, data were expressed as means ± SD, *$P < 0.05$, **$P < 0.01$, ***$P < 0.001$, ****$P < 0.0001$). Source data are provided as a Source Data file.

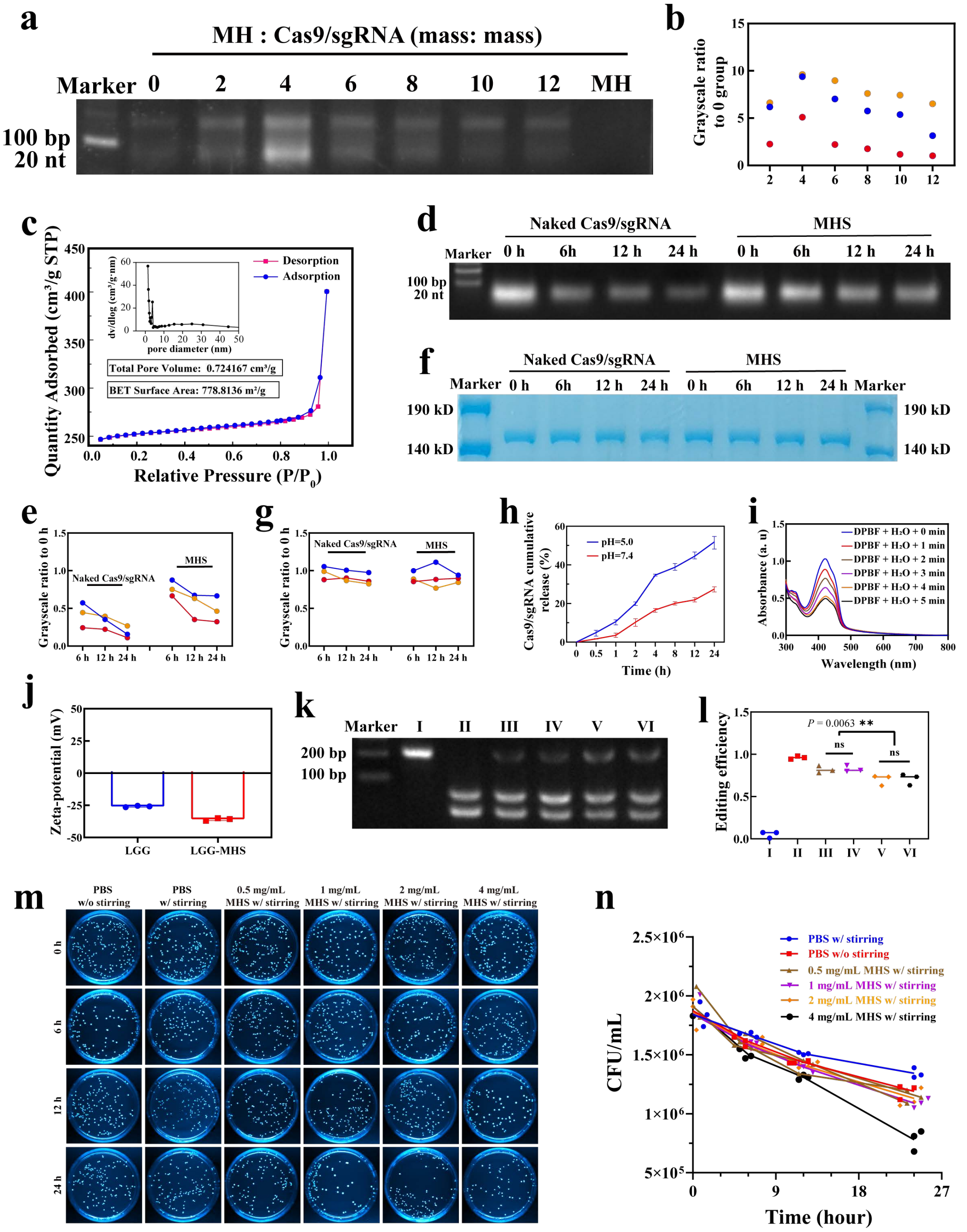

Supplementary Figure 2. (a) Agarose gel electrophoresis and (b) corresponding quantitative analysis of MHS nanoparticles at different MH/sgRNA ratios after incubation with serum (10% volume) for 6 h. Group 0 *i.e.* naked Cas9/sgRNA. (*n* = 3 independent experiments) (c) N_2 adsorption-desorption isotherms and of MH. The inset shows its corresponding total pore volume and specific surface area. (d) Agarose gel electrophoresis and (e) corresponding quantitative analysis to evaluate the serum stability of naked Cas9/sgRNA and Cas9/sgRNA reconstituted from MHS (*n* = 3 independent experiments). (f) SDS-PAGE and (g) corresponding quantitative analysis to evaluate the serum stability of Cas9/sgRNA and Cas9/sgRNA reconstituted from MHS (*n* = 3 independent experiments). (h) Release of Cas9/sgRNA from MHS. (*n* = 3 independent samples,

data were expressed as means ± SD). (i) UV-vis absorption spectra of 1,3-diphenylisobenzofuran (DPBF) upon US irradiation for prolonged durations. (j) Zeta- potential of LGG and LGG-MHS (n = 3 independent samples, data were expressed as means ± SD). (k) Agarose gel electrophoresis and (l) corresponding quantitative analysis of the activity of CRISPR/Cas9 nanosystem under different states, including I (DNA Only), II (Cas9/sgRNA + DNA), III (MHS + DNA), IV (MHS + US + DNA), V (LGG-MHS + DNA), VI (LGG-MHS + US + DNA) (n = 3 biologically independent experiments). Statistical differences were calculated using two-tailed unpaired Student's t-test for comparisons between two groups, ordinary one-way ANOVA for comparisons more than two groups. *$P < 0.05$, **$P < 0.01$, ***$P < 0.001$, ****$P < 0.0001$. (m) Representative photographs and (n) corresponding CFU quantitative of MRS agar plates of bacterial activity with various concentrations of MHS in a different time (0, 2, 6, 12 and 24 h) (n = 3 independent samples). The experiments for **a**, **c**, **d**, **f**, **k** and **i** were repeated three times independently with similar results. Source data are provided as a Source Data file.

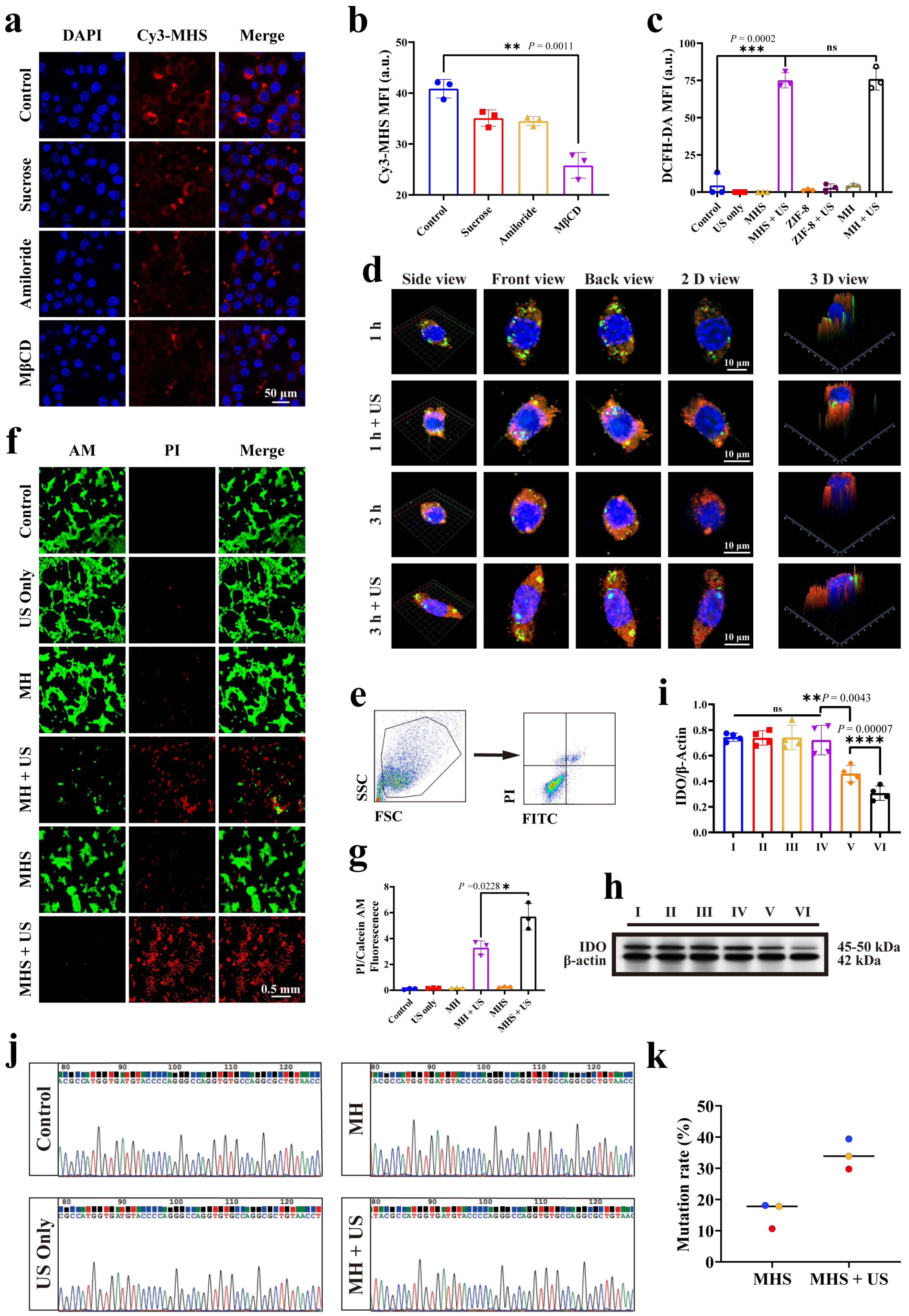

Supplementary Figure 3. (a) CLSM images and (b) the corresponding mean fluorescence intensity analysis of cellular uptake of Cy3-labeled MHS by 4T1 cancer-cell line after coincubation with different inhibitors (n = 3 biologically independent samples) (c) Fluorescence intensity of CLSM images of 4T1 cells with different treatments (n = 3 biologically independent samples) (d) Z-stack

CLSM images of 4T1 cells cultured with Cy5.5-labeled MHS nanosystem upon US irradiation for 1 and 3 h at 37 °C. The cell nuclei were stained with DAPI (blue), endo/lysosomes were stained with LysoTracker Green (green), and MHS was labeled with Cy5.5 (red) (e) Gating strategies for isolating $PI^{+}FITC^{+}$ 4T1 cells. (f) CLSM observation and (g) corresponding PI/ calcein-AM fluorescence intensity ratio of 4T1 cells stained by calcein-AM (green) and PI (red) after various treatments (n = 3 biologically independent samples). (h) Western Blot and (i) corresponding quantitative analysis of IFN-γ-stimulated 4T1 cells with various treatments. (I = control, II = US only, III = MH, IV = MH + US, V = MHS, VI = MHS + US) (n = 4 biologically independent experiments). (j) *In vitro* gene-editing efficiency in 4T1 cells. DNA sequencing of *IDO1* after various treatments (The representative data of deep sequencing from three independent experiments). (k) Corresponding quantitative analysis of T7E I cleavage after 4T1 cells with MHS and MHS + US treatment (n = 3 biologically independent experiments, data were expressed as median). Representative images of three biologically independent samples from each group is shown in **a**, **d** and **f**, and four times each experiment was repeated independently with similar results for **h**. Statistical differences of **b**, **c**, **g** and **i**, were calculated using two-tailed unpaired Student's t-test for comparisons between two groups, Dunnett's multiple comparisons post test for comparisons more than two groups containing group Control, data were expressed as means ± SD. *P < 0.05, **P < 0.01, ***P < 0.001, ****P < 0.0001. Source data are provided as a Source Data file.

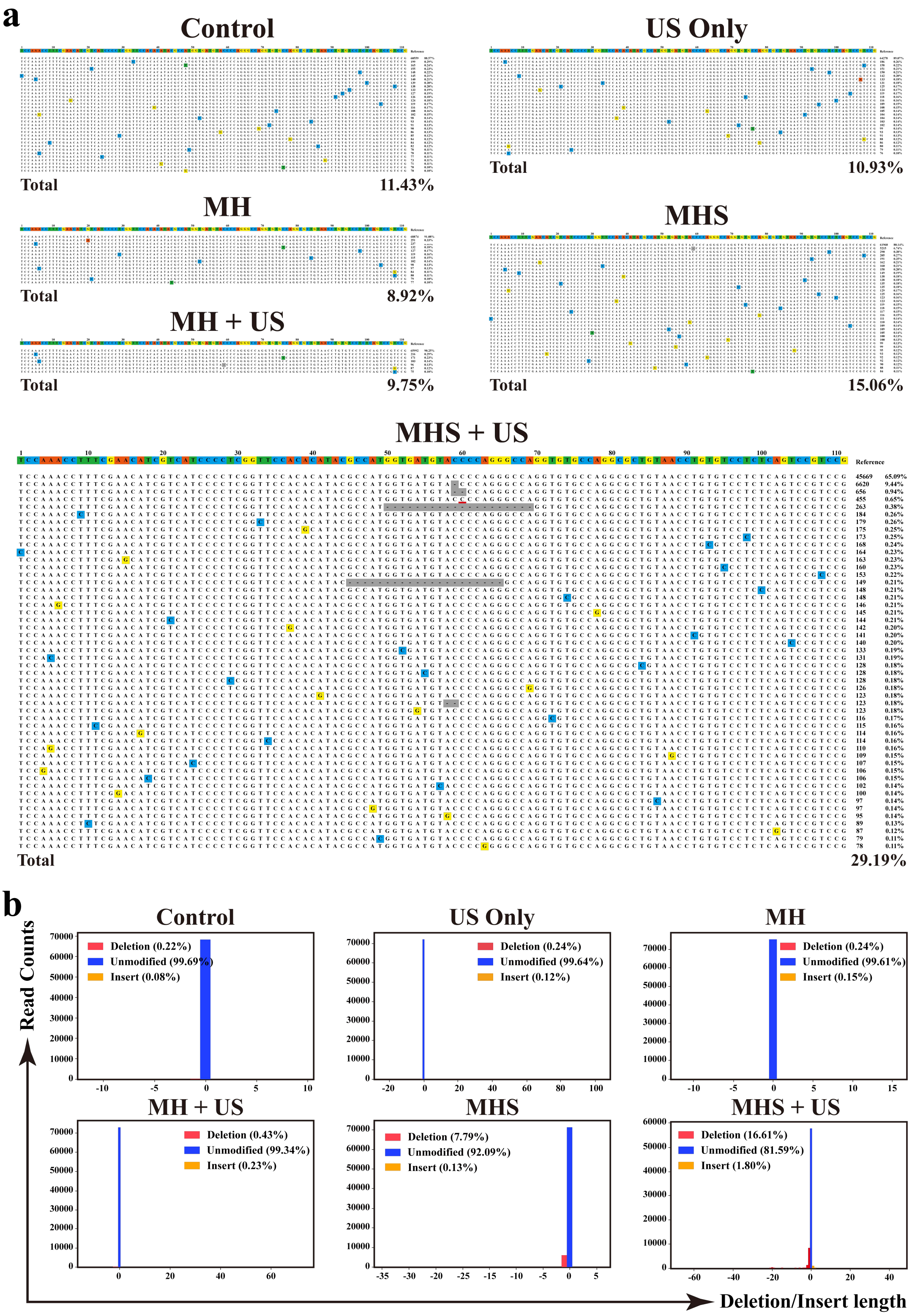

Supplementary Figure 4. (a) Deep sequencing for targeted disruption of *IDO1* locus in control, US only, MH, MH + US, MHS and MHS + US. (b) Nucleotide deletion and insert distribution around the cut site of *IDO1* locus in control, US only, MH, MH + US, MHS and MHS + US. The experiments for **a** and **b** were repeated three times independently with similar results.

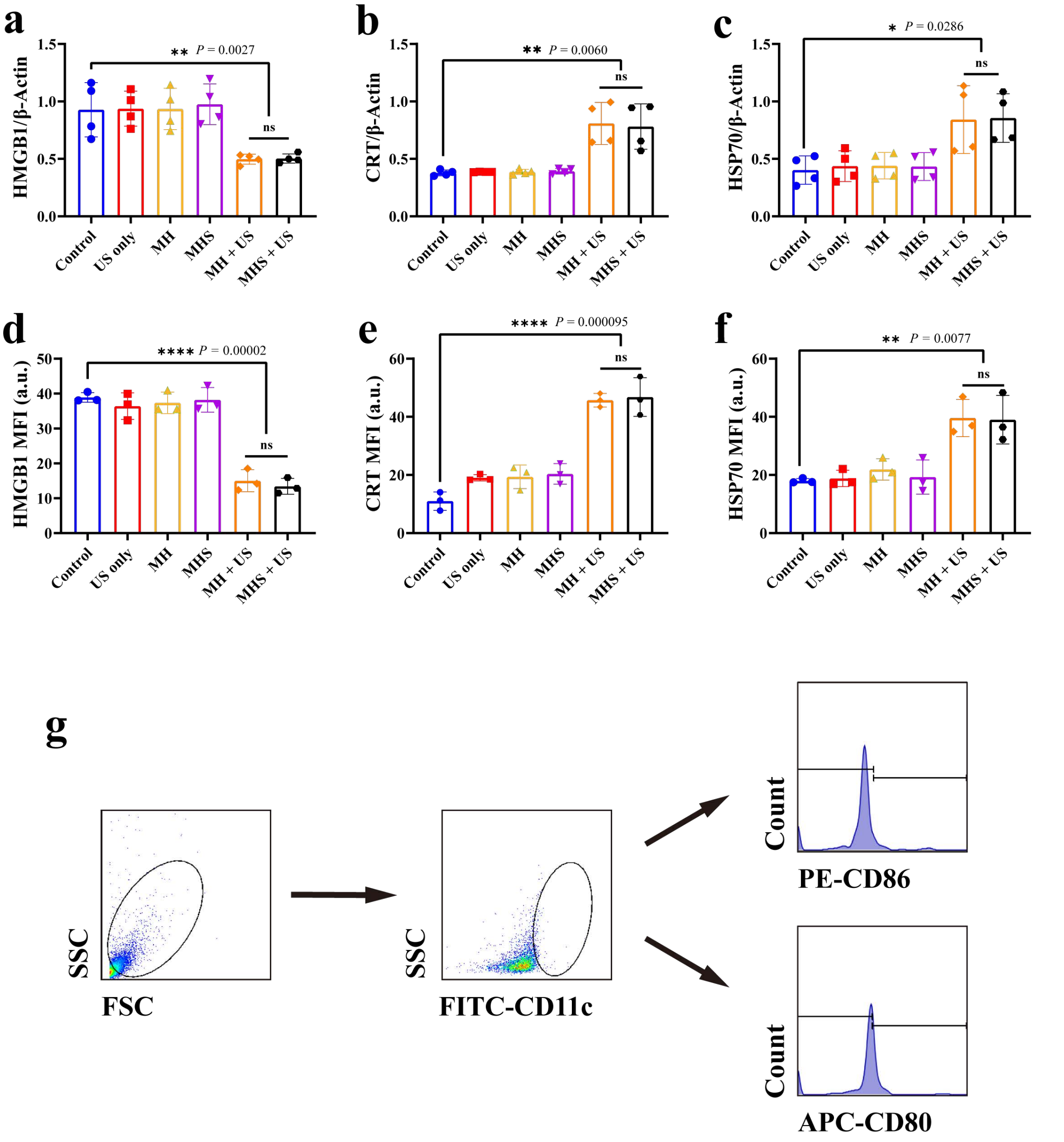

Supplementary Figure 5. (a-c) The quantitative analysis of HMGB1, CRT and HSP70 on Western Blot (*n* = 4 biologically independent experiments, data were expressed as means ± SD). Statistical differences were calculated using two-tailed paired Student's t-test between two groups, Dunnett's multiple comparisons post test for comparisons more than two groups containing group Control. *P < 0.05, **P < 0.01, ***P < 0.001, ****P < 0.0001. (d-f) Fluorescence intensity of HMGB1, CRT and HSP70 on CLSM (*n* = 3 biologically independent samples, data were expressed as means ± SD). Statistical differences were calculated using two-tailed unpaired Student's t-test between two groups, Dunnett's multiple comparisons post test for comparisons more than two groups containing group Control. *P < 0.05, **P < 0.01, ***P < 0.001, ****P < 0.0001. (g) Gating strategies for isolating $CD80^+CD86^+$ mature DCs. Source data are provided as a Source Data file.

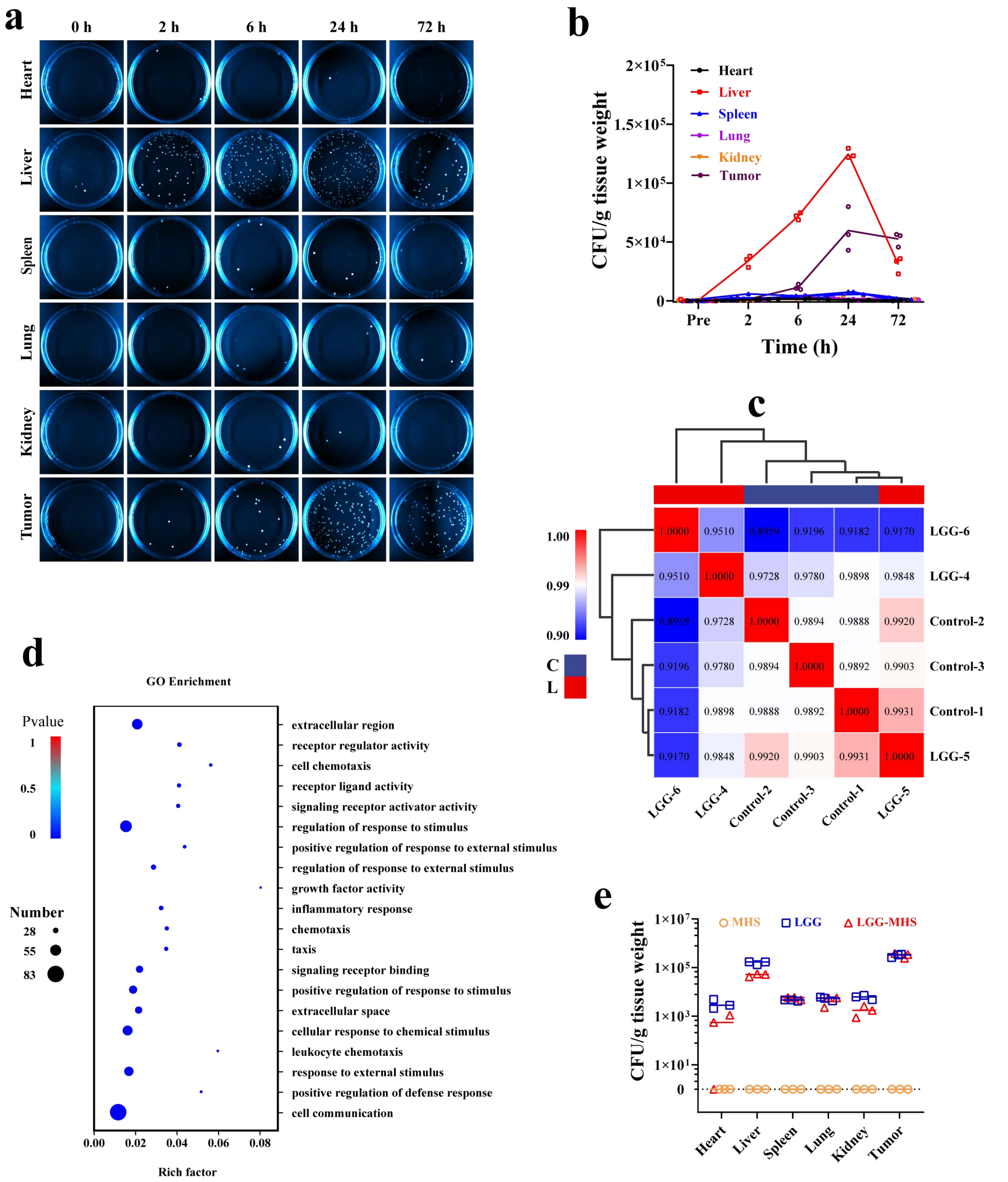

Supplementary Figure 6. (a) Representative photographs of MRS agar plates and (b) corresponding quantitative analysis of bacterial colonization in various organs and tumor of 4T1-bearing mice in a different time (0, 2, 6, 24, and 72 h) (n = 3 biologically independent samples). (c) Sample correlation test of genes alteration with or without LGG treatment. (d) GO analysis of differential gene expression profiles based on RNAseq after LGG treatment (n = 3 mice per group). Statistical difference was calculated using Fisher's exact test. (e) Corresponding quantitative analysis of bacterial colonization in various organs harvested from 4T1-bearing mice at various time points after injection of MHS, LGG and LGG-MHS on solid MRS agar plates (n = 3 biologically independent samples, data were expressed as median). Source data are provided as a Source Data file.

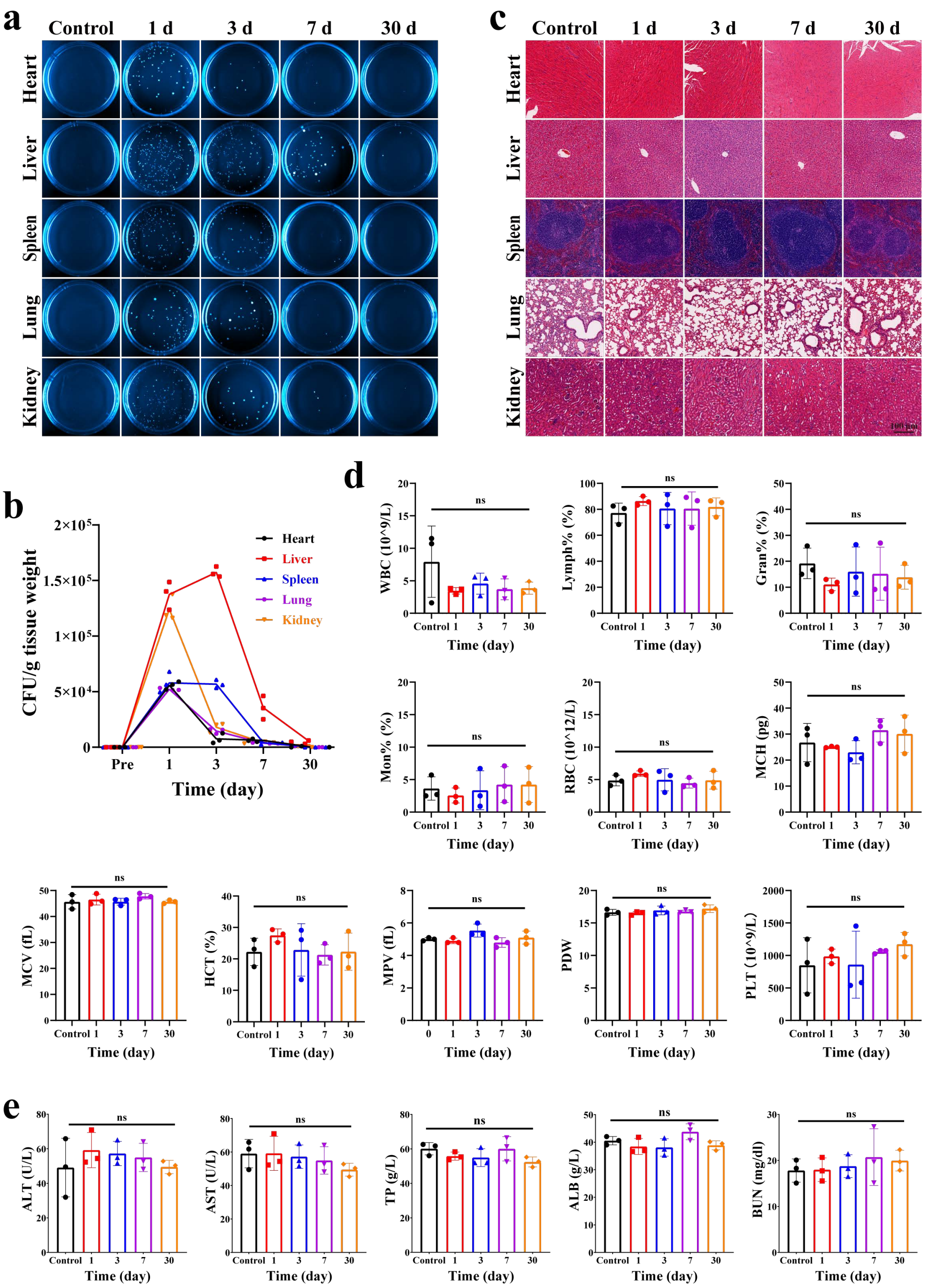

Supplementary Figure 7. (a) Representative photographs and (b) corresponding CFU count analysis of MRS agar plates of bacterial colonization in various organs of healthy mice in a month (1, 3, 7 and 30 days) (n = 3 biologically independent samples), Control *i.e.* without any treatment. (c) HE staining of histological sections of various organs in healthy mice after receiving LGG-MHS injection within one month (1, 3, 7 and 30 days), Control *i.e.* without any treatment. (d) *In vivo* hematological indices. Hematological assays of mice at 1, 3, 7 and 30 days after LGG-MHS injection (n = 3 biologically independent samples). Control *i.e.* without any treatment. (e) *In vivo* liver and kidney function index. Hematological assays of mice at 1, 3, 7 and 30 days after LGG-MHS injection (n = 3 biologically independent samples). Control *i.e.* without any treatment. A representative image of 3 biologically independent samples from each group is shown in **a** and **c**.

Statistical differences for **d** and **e** were calculated using Dunnett's multiple comparisons post test, data were expressed as means ± SD. *$P < 0.05$, **$P < 0.01$, ***$P < 0.001$, ****$P < 0.0001$. Source data are provided as a Source Data file.

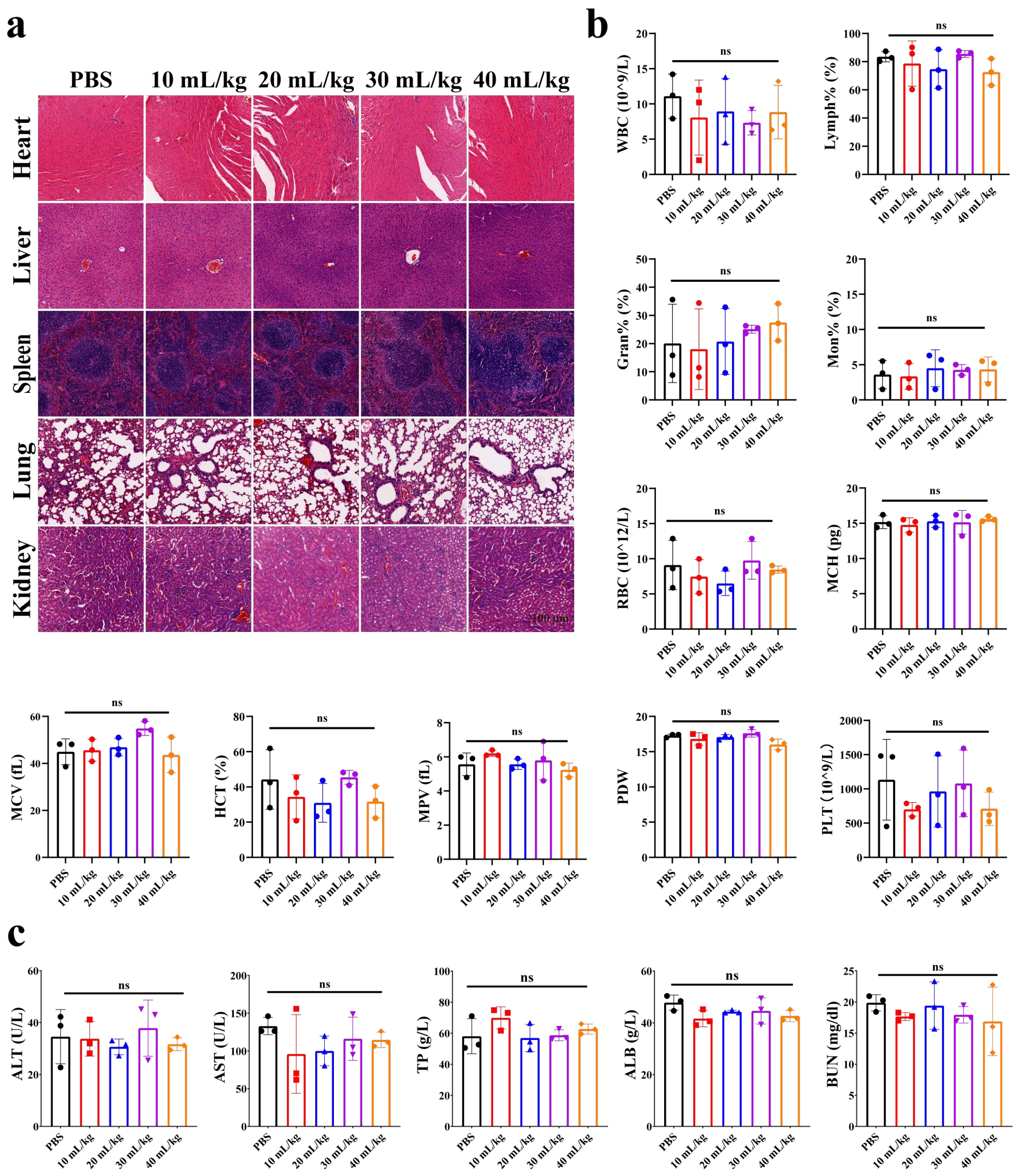

Supplementary Figure 8. (a) HE staining of histological sections of healthy mice treated with different doses of LGG-MHS (PBS, 10 ml/kg, 20 ml/kg, 30 ml/kg, 40 ml/kg. 1 mL LGG-MHS = 1 × 10^7 LGG, 1 mg MHS) and subjected to US irradiation of each organ (The representative imaging from 3 independent samples). (b) *In vivo* hematological indices. Hematological assays of healthy mice treated with different doses of LGG-MHS (PBS, 10 ml/kg, 20 ml/kg, 30 ml/kg, 40 ml/kg. 1 mL LGG-MHS = 1 × 10^7 LGG, 1 mg MHS). (n = 3 biologically independent samples). (c) *In vivo* liver and kidney function index. Hematological assays of mice healthy mice treated with different doses of LGG-MHS (PBS, 10 ml/kg, 20 ml/kg, 30 ml/kg, 40 ml/kg. 1 mL LGG-MHS = 1 × 10^7 LGG, 1 mg MHS) (n = 3 biologically independent animals). Statistical differences for **b** and **c** were calculated using Dunnett's multiple comparisons post test, data were expressed as means ± SD. *P < 0.05, **P < 0.01, ***P < 0.001, ****P < 0.0001. Source data are provided as a Source Data file.

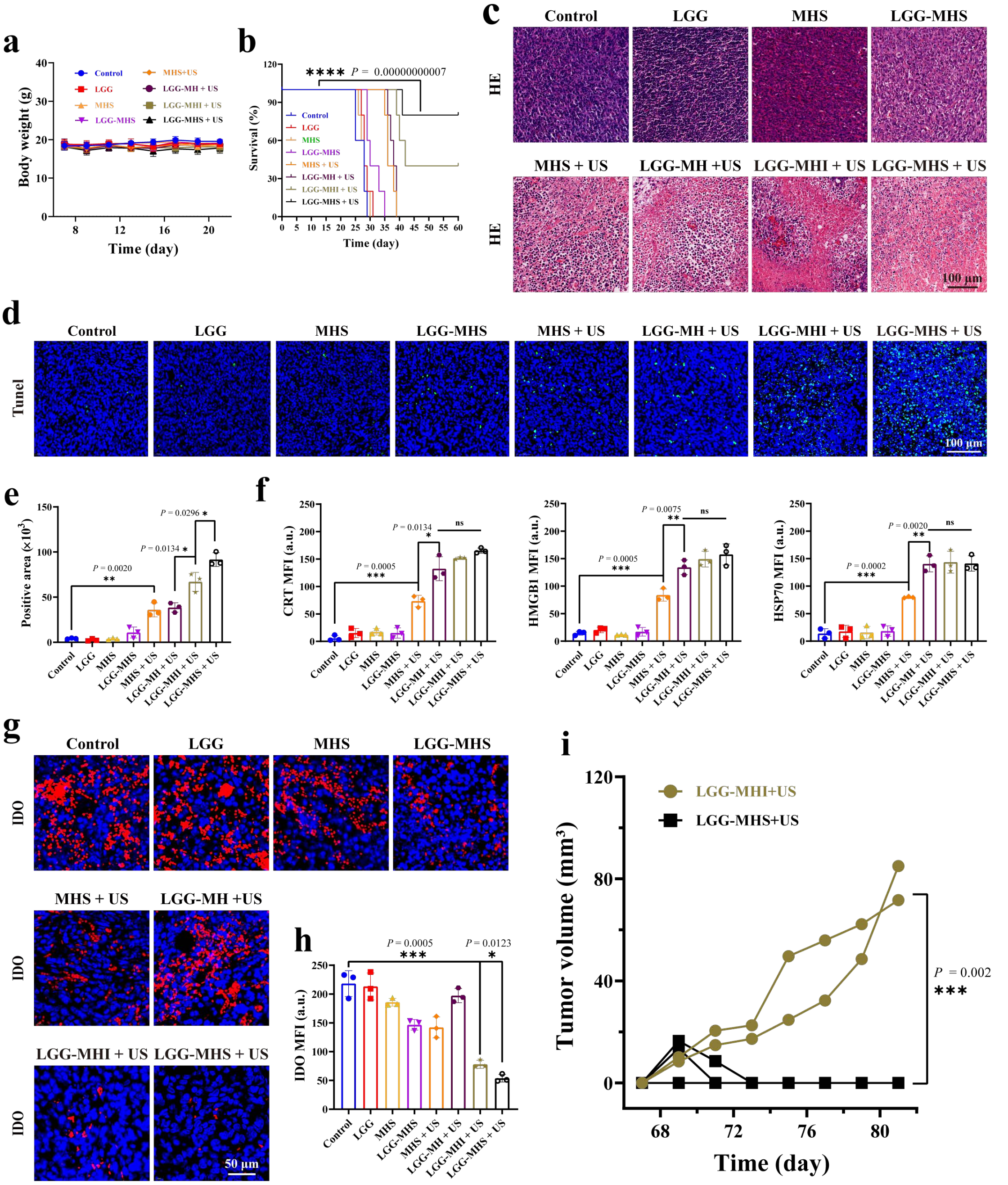

Supplementary Figure 9. (a) Body weight and (b) survival curves of 4T1-tumor-bearing mice with different treatment (control, US only, MH, MH + US, MHS, and MHS + US) (n = 5 mice per group, data of mice body weight were expressed as means ± SD. Statistical differences of survival were calculated using Log-rank test). (c) HE staining of primary tumor histologic sections after different treatments. (d) Immunofluorescence images and (e) corresponding of TUNEL assay in primary tumor tissue after different treatments. DAPI was used to stain the nucleus of the cell (blue) (n = 3 biologically independent samples). (f) Corresponding fluorescence intensity of specific proteins expression after DAMPs (HMGB1, CRT, and HSP70) from tumor tissue. (n = 3 biologically independent samples). (g) Images and (h) corresponding fluorescence intensity of IDO immunofluorescence staining in primary tumors of 4T1 tumor-bearing mice after various treatments. DAPI was used to stain the nucleus of the cell (blue), and the IDO was stained with anti-IDO

antibodies (red) (n = 3 biologically independent samples. (i) Average tumor growth curves after being treated by re-challenge. ($n_{\text{LGG-MHI + US}}$ = 2 biologically independent animals, $n_{\text{LGG-MHS + US}}$ = 4 biologically independent animals). Statistical differences were calculated using two-way ANOVA with the Geisser-Greehouse correction, match values are stacked into a subcolumn. *$P < 0.05$, **$P < 0.01$, ***$P < 0.001$, ****$P < 0.0001$. A representative image of three biologically independent samples from each group is shown in **c**, **d** and **g**. Statistical differences for **e**, **f** and **h** were calculated using two-tailed unpaired Student's t-test between two groups, ordinary one-way ANOVA for comparisons more than two groups, data were expressed as means ± SD. *$P < 0.05$, **$P < 0.01$, ***$P < 0.001$, ****$P < 0.0001$. Source data are provided as a Source Data file.

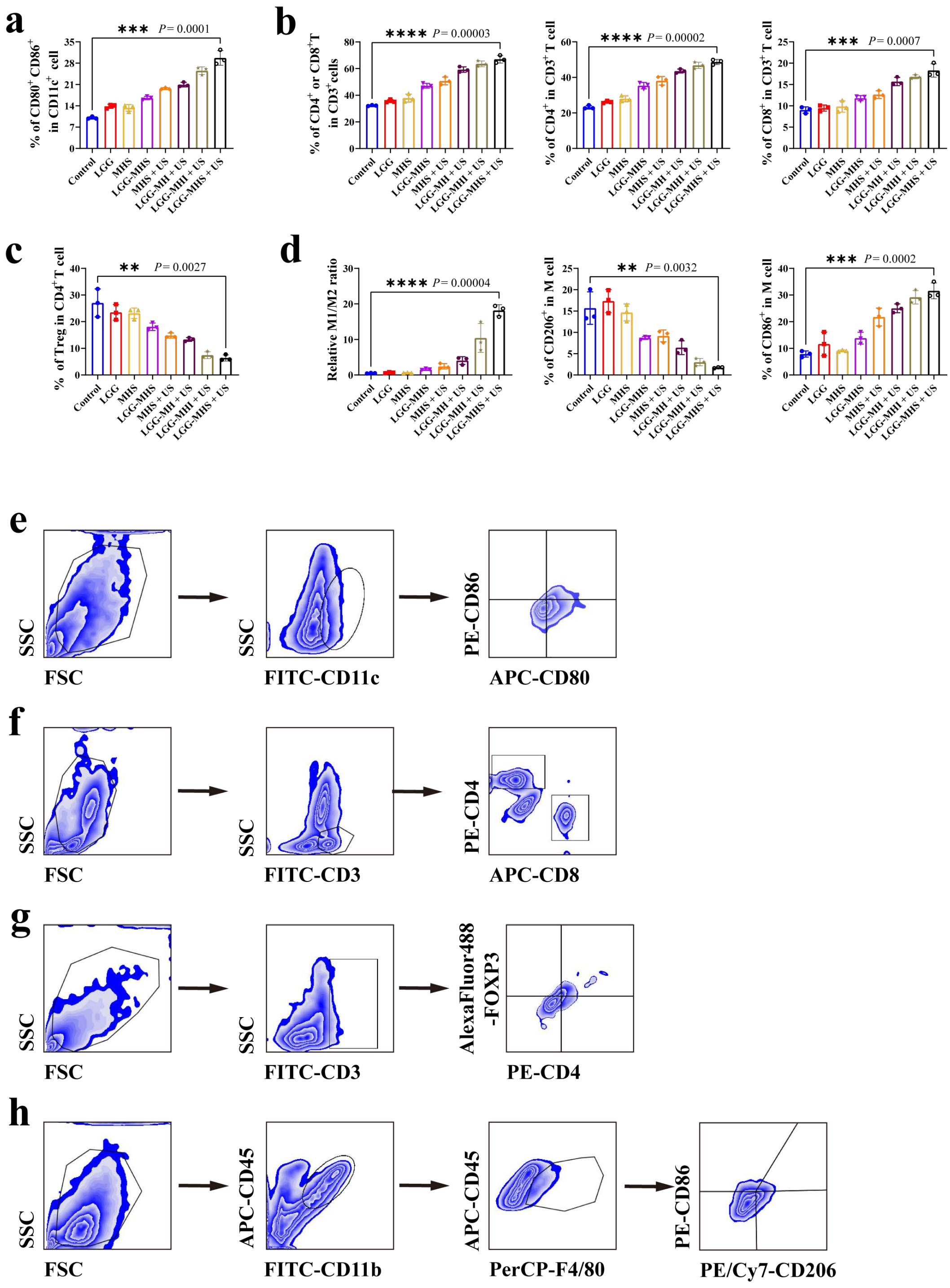

Supplementary Figure 10. (a) The quantitative analysis of mature DCs in tumor tissue after 24 h after the first different treatments (n = 3 biologically independent samples). (b) The quantification of $CD4^+$ and $CD8^+$ T cells in the spleen after 24 h after the first different treatments (n = 3 biologically independent samples). (c) The quantitative analysis of Tregs in primary tumor tissue after 24 h after the first different treatments (n = 3 biologically independent samples). (d) The quantitative analysis of M1, M2 and M1/M2 macrophages ratio in spleen after 24 h after the first different treatments (n = 3 biologically independent samples). (e) Gating strategies for isolating $CD80^+CD86^+$ mature DCs from tumor tissue. (f) Gating strategies for isolating $CD4^+$ and $CD8^+$ T cells from spleen tissue. (g) Gating strategies for isolating Tregs from tumor tissue. (h) Gating

strategies for isolating M2 macrophages from spleen tissue. Statistical differences were calculated using two-tailed unpaired Student's t-test in **a**, **b**, **c** and **d**, data were expressed as means ± SD. $*P < 0.05$, $**P < 0.01$, $***P < 0.001$, $****P < 0.0001$. Source data are provided as a Source Data file.

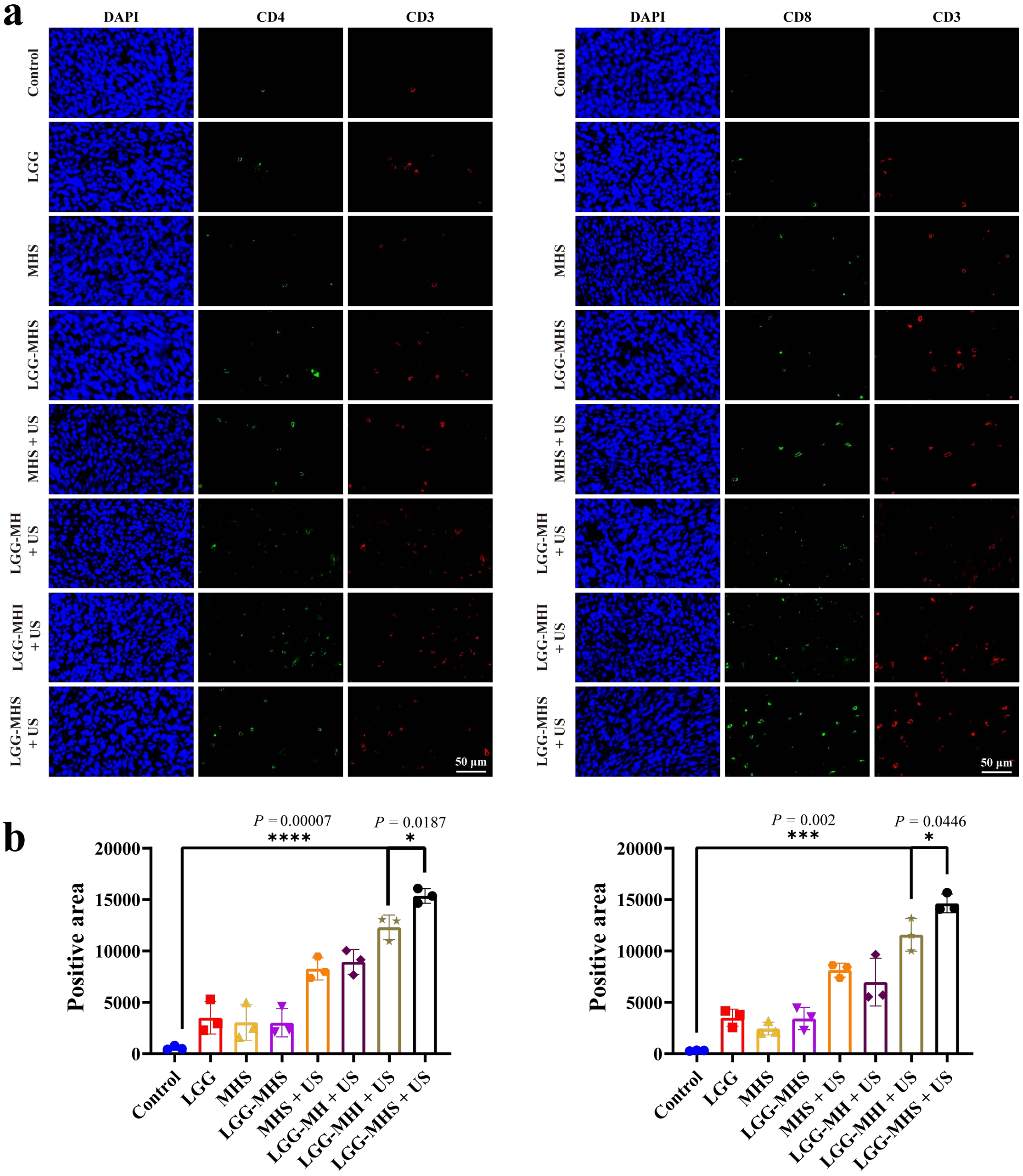

Supplementary Figure 11. (a) Different channels of immunofluorescence images and (b) corresponding positive area quantification of $CD3^+CD4^+$ and $CD3^+CD8^+$ proliferating CTLs in primary 4T1 tumor tissue sections after different treatments (control, LGG, MHS, LGG-MHS, MHS+US, LGG-MH+US, LGG-MHI+US and LGG-MHS+US (n = 3 biologically independent samples, data were expressed as means ± SD). Statistical differences were calculated using two-tailed unpaired Student's t-test. *$P < 0.05$, **$P < 0.01$, ***$P < 0.001$, ****$P < 0.0001$. Source data are provided as a Source Data file.

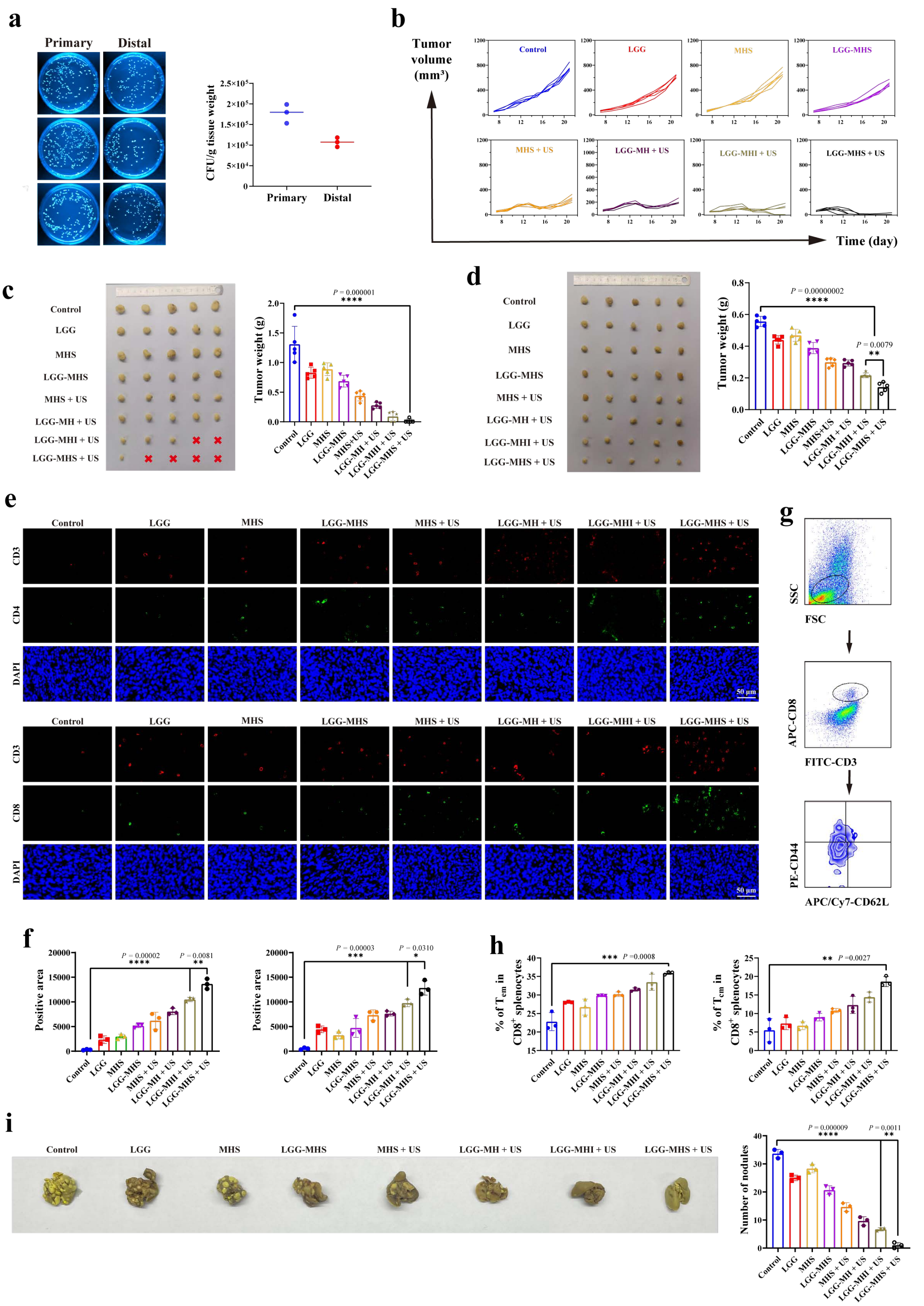

Supplementary Figure 12. (a) Representative photographs of MRS agar plates and (b) corresponding quantitative analysis of bacterial colonization in primary and distal tumor of 4T1-bearing mice (n = 3 biologically independent samples, data were expressed as median). (c) Individual tumor growth curves of primary tumor after being untreated, treated by LGG, MHS, LGG-MHS, MHS + US, LGG-MH + US, LGG-MHI + US and LGG-MHS + US (n = 5 mice per group). (c) Digital images and weight statistics of primary and (d) distal tumors of 4T1 tumor-

bearing mice at the 21th day after different treatments (control, LGG, MHS, LGG-MHS, MHS + US, LGG-MH + US, LGG-MHI + US and LGG-MHS + US) (*n* = 5 biologically independent samples). (e) Different channels of immunofluorescence images and (f) corresponding positive area quantification of $CD3^+CD4^+$ and $CD3+CD8^+$ proliferating CTLs in distant 4T1 tumor tissue sections after various treatments, including control, LGG, MHS, LGG-MHS, MHS + US, LGG-MH + US, LGG-MHI + US and LGG-MHS + US (*n* = 3 biologically independent samples). (g) Gating strategies for isolating T cells (CD3+CD8+CD44+CD62L−) (Tem) and (CD3+CD8+CD44+CD62L+) (Tcm). (h) Corresponding quantification of the effector memory T cells ($CD3^+CD8^+CD44^+CD62L^-$) (Tem) and ($CD3^+CD8^+CD44^+CD62L^+$) (Tcm) in the spleen after 24 h after the first different treatments (*n* = 3 biologically independent samples). (i) Representative photographs and counts of the number of lung metastatic nodules after various treatment (control, LGG, MHS, LGG-MHS, MHS + US, LGG-MH + US, LGG-MHI + US and LGG-MHS + US) (*n* = 3 biologically independent samples). Representative images of three biologically independent samples from each group is shown in **a**, **e** and **i**. Statistical differences for **c**, **d**, **f**, **h** and **i** were calculated using two-tailed unpaired Student's t-test for comparisons between two groups, ordinary one-way ANOVA for comparisons more than two groups, data were expressed as means ± SD. $^*P < 0.05$, $^{**}P < 0.01$, $^{***}P < 0.001$, $^{****}P < 0.0001$. Source data are provided as a Source Data file.

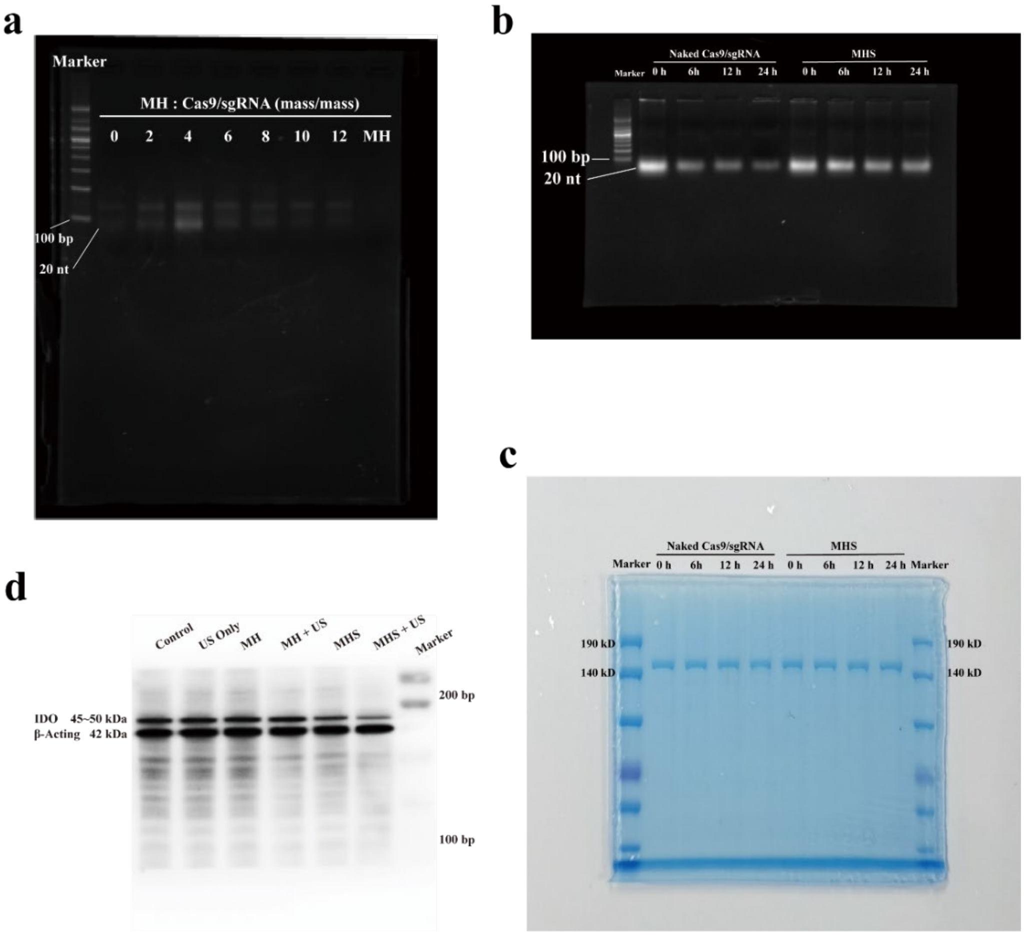

Supplementary Figure 13. (a) Uncropped scans of gels in Supplementary Figures 2a. (b) Uncropped scans of gels in Supplementary Figures 2d. (c) Uncropped scans of gels in Supplementary Figures 2f. (d) Uncropped scans of blots in Supplementary Figures 3g.

Supplementary Table

Supplementary Table 1: Corresponding atomic fraction of **Fig. 2d.**

	Zn (%)	P (%)	N (%)	C (%)
ZIF-8	1.09	0.01	3.89	95.01
MH	0.95	0.01	2.67	96.37
MHS	3.88	0.56	4.95	90.61
