# Supplementary material for: Design of a self-driven probiotic-CRISPR/Cas9 nanosystem for sono-immunometabolic cancer therapy
Source: Nat Commun. 2022 Dec 22;13:7903. doi: 10.1038/s41467-022-35580-z (PMC9780327; doi:10.1038/s41467-022-35580-z)
Supplement: Supplementary file 3 — Reporting Summary [file 41467_2022_35580_MOESM3_ESM.pdf]

nature portfolio

Corresponding author(s): Haohao Yin; Huixiong Xu

Last updated by author(s): Nov 28, 2022

Reporting Summary

Nature Portfolio wishes to improve the reproducibility of the work that we publish. This form provides structure for consistency and transparency in reporting. For further information on Nature Portfolio policies, see our Editorial Policies and the Editorial Policy Checklist.

Statistics

For all statistical analyses, confirm that the following items are present in the figure legend, table legend, main text, or Methods section.

n/a	Confirmed	
☐	☒	The exact sample size (*n*) for each experimental group/condition, given as a discrete number and unit of measurement
☐	☒	A statement on whether measurements were taken from distinct samples or whether the same sample was measured repeatedly
☐	☒	The statistical test(s) used AND whether they are one- or two-sided<br>*Only common tests should be described solely by name; describe more complex techniques in the Methods section.*
☐	☒	A description of all covariates tested
☐	☒	A description of any assumptions or corrections, such as tests of normality and adjustment for multiple comparisons
☐	☒	A full description of the statistical parameters including central tendency (e.g. means) or other basic estimates (e.g. regression coefficient) AND variation (e.g. standard deviation) or associated estimates of uncertainty (e.g. confidence intervals)
☐	☒	For null hypothesis testing, the test statistic (e.g. *F*, *t*, *r*) with confidence intervals, effect sizes, degrees of freedom and *P* value noted<br>*Give P values as exact values whenever suitable.*
☒	☐	For Bayesian analysis, information on the choice of priors and Markov chain Monte Carlo settings
☒	☐	For hierarchical and complex designs, identification of the appropriate level for tests and full reporting of outcomes
☒	☐	Estimates of effect sizes (e.g. Cohen's *d*, Pearson's *r*), indicating how they were calculated

Our web collection on statistics for biologists contains articles on many of the points above.

Software and code

Policy information about availability of computer code

Data collection	The specific surface area and pore size of the composite nanoparticles were measured by nitrogen adsorption technique in a Micrometitics ASAP 2460 system at -195.850 °C. TEM and corresponding element mapping were adopted for the observation of the microstructure and component of nanosystem (FEI Tecnai G2 F20 operated at 200 kV and JEOL). Particle size and zeta potential were determined by Zetasizer Nanoseries (ZTS1240, Malvern Panalytical Ltd.). UV–vis spectra was acquired by UV-vis-NIR spectrophotometer (PE Lambda 950). Nikon bio-microscope (CI-L) was used to observe the fluorescent sections.
Data analysis	GraphPad Prism (version 9.0.0, GraphPad Software, San Diego, California, USA) was employed to calculate all statistical analyses. All flowcytometry data were analyzed on FlowJoTM software package (version 10.5.2, BD Life Sciences, Ashland, Oregon, USA). Living imaging software CleVue (version 3.1.3.2054, Vieworks Co., Ltd, Anyang-si, Gyeonggi-do, Republic of KOREA) was used to analyse bioluminescent and fluorescent images.

For manuscripts utilizing custom algorithms or software that are central to the research but not yet described in published literature, software must be made available to editors and reviewers. We strongly encourage code deposition in a community repository (e.g. GitHub). See the Nature Portfolio guidelines for submitting code & software for further information.

Data

Policy information about availability of data

All manuscripts must include a data availability statement. This statement should provide the following information, where applicable:

- Accession codes, unique identifiers, or web links for publicly available datasets
- A description of any restrictions on data availability
- For clinical datasets or third party data, please ensure that the statement adheres to our policy

The data that support the finding of this study are available within the Article, Supplementary Information or Source Data file. The RNA-sequencing data generated in this study have been deposited in the Genome Sequence Archive (GSA) database under accession code CRA007764 [https://ngdc.cncb.ac.cn/search/?dbId=gsa&q=+CRA007764]. Source data are provided with this paper.

Human research participants

Policy information about studies involving human research participants and Sex and Gender in Research.

Reporting on sex and gender	N/A
Population characteristics	N/A
Recruitment	N/A
Ethics oversight	N/A

Note that full information on the approval of the study protocol must also be provided in the manuscript.

Field-specific reporting

Please select the one below that is the best fit for your research. If you are not sure, read the appropriate sections before making your selection.

[x] Life sciences [ ] Behavioural & social sciences [ ] Ecological, evolutionary & environmental sciences

For a reference copy of the document with all sections, see nature.com/documents/nr-reporting-summary-flat.pdf

Life sciences study design

All studies must disclose on these points even when the disclosure is negative.

Sample size	Althoughno samplesize calculation was performed, sample sizes (at least three animals per treatment group) represents the minimum numberanimals needed to reach statistical significance (p <0.05) between experimenta groups. Meanwhile, sample sizes for the in vivoexperiments are similarto thosegenerally employed and accepted in the field (Nat Com mun 12, 3187 (2021); Nat Nanotechnol 16, 1271-1280(2021))and were sufficient to supportour conclusions with statistical significance. Samp ble sizes for the in vitro experiments are also based on previous work (Nat Nanotechnol 16,538-548(2021))
Data exclusions	No data were excluded.
Replication	Experiment were repeated and experimental findings were reproducible. Details of experimental replicates are given in the figure legends.
Randomization	All experimental samples or models were allocated randomly to each group.
Blinding	The investigator were not blinded for most of experiments since the experimental design, execution and data analysis were performed by the same person. Bioluminescence imaging were conducted by an independent operator, w no was unaware of tne treatment conaitions.

Reporting for specific materials, systems and methods

We require information from authors about some types of materials, experimental systems and methods used in many studies. Here, indicate whether each material, system or method listed is relevant to your study. If you are not sure if a list item applies to your research, read the appropriate section before selecting a response.

Materials & experimental systems

n/a	Involved in the study	
☐	☒	Antibodies
☐	☐	Eukaryotic cell lines
☒	☐	Palaeontology and archaeology
☐	☒	Animals and other organisms
☒	☐	Clinical data
☒	☐	Dual use research of concern

Methods

n/a	Involved in the study	
☒	☐	ChIP-seq
☐	☒	Flow cytometry
☒	☐	MRI-based neuroimaging

Antibodies

Antibodies used

(1) IDO (D5J4E™) Rabbit mAb (Cell Signaling Technology, Cat# 86630).
(2) HMGB1 Anti-Rabbit Antibody (Affinity Biosciences, Cat# AF7020).
(3) CRT Anti-Rabbit Antibody (Affinity Biosciences, Cat# DF10202).
(4) HSP70 Anti-Rabbit Antibody (Affinity Biosciences, Cat# AF5466).
(5) β-actin Anti- Rabbit Antibody (Abcam, Cat# ab8227).
(6) Goat Anti-Rabbit IgG H&L (Alexa Fluor® 488) (Abcam, Cat# ab150077).
(7) Goat Anti-Rabbit IgG H&L (Alexa Fluor® 555) (Abcam, Cat# ab150078).
(8) FITC anti-mouse CD11c Antibody (Biolegend, Cat# 117306, Clone No. N418).
(9) PE anti-mouse CD86 Antibody (Biolegend, Cat# 105007, Clone No.GL-1).
(10) APC anti-mouse CD80 Antibody (Biolegend, Cat# 104714, Clone No.16-10A1).
(11) FITC anti-mouse CD3 (Biolegend, Cat# 100203, Clone No.17A2).
(12) PE anti-mouse CD4 (Biolegend, Cat# 100407GK1.5).
(13) APC anti-mouse CD8a (Biolegend, Cat# 100712, Clone No.53-6.7).
(14) AlexaFluor488 anti-mouse FOXP3 (Biolegend, Cat# 320012, Clone No.150D).
(15) FITC anti-mouse/human CD11b Antibody (Biolegend, Cat# 101205, Clone No. M1/70).
(16) APC anti-mouse CD45 (Biolegend, Cat# 103112, Clone No.30-F11).
(17) PerCP anti-mouse F4/80 Antibody (Biolegend, Cat# 123126, Clone No.BM8).
(18) PE/Cyanine7 anti-mouse CD206 (MMR) Antibody (Bioegend, Cat# 141720, Clone No.C068C2).
(19) PE anti-mouse/human CD44 Antibody (Bioegend, Cat# 103007, Clone No.IM7).
(20) APC/Cyanine7 anti-mouse CD62L Antibody (Bioegend, Cat# 104428, Clone No.MEL-14).

Validation

(1) IDO (D5J4E™) Rabbit mAb. https://www.cellsignal.cn/products/primary-antibodies/ido-d5j4e-rabbit-mab/86630?site-search-type=Products&N=4294956287&Ntt=86630&fromPage=plp&_requestid=4893015
(2) HMGB1 Anti-Rabbit Antibody. https://www.affbiotech.cn/goods-2072-AF7020-HMGB1_Antibody.html
(3) CRT Anti-Rabbit Antibody. https://www.affbiotech.cn/goods-11044-DF10202-CALCR_Antibody.html
(4) HSP70 Anti-Rabbit Antibody. https://www.affbiotech.cn/goods-4772-AF5466-HSP70_Antibody.html
(5) β-actin Anti- Rabbit Antibody. https://www.abcam.cn/beta-actin-antibody-ab8227.html
(6) Goat Anti-Rabbit IgG H&L (Alexa Fluor® 488). https://www.abcam.cn/goat-rabbit-igg-hl-alexa-fluor-488-ab150077.html
(7) Goat Anti-Rabbit IgG H&L (Alexa Fluor® 555). https://www.abcam.cn/goat-rabbit-igg-hl-alexa-fluor-555-ab150078.html
(8) FITC anti-mouse CD11c Antibody. https://www.biolegend.com/en-us/products/fitc-anti-mouse-cd11c-antibody-1815
(9) PE anti-mouse CD86 Antibody. https://www.biolegend.com/en-us/products/pe-anti-mouse-cd86-antibody-256
(10) APC anti-mouse CD80 Antibody. https://www.biolegend.com/en-us/products/apc-anti-mouse-cd80-antibody-2340
(11) FITC anti-mouse CD3. https://www.biolegend.com/en-us/products/fitc-anti-mouse-cd3-antibody-45
(12) PE anti-mouse CD4. https://www.biolegend.com/en-us/products/pe-anti-mouse-cd4-antibody-250
(13) APC anti-mouse CD8a. https://www.biolegend.com/en-us/products/apc-anti-mouse-cd8a-antibody-150
(14) AlexaFluor488 anti-mouse FOXP3. https://www.biolegend.com/en-us/products/alexa-fluor-488-anti-mouse-rat-human-foxp3-antibody-2891
(15) FITC anti-mouse/human CD11b Antibody. https://www.biolegend.com/en-us/products/fitc-anti-mouse-human-cd11b-antibody-347
(16) APC anti-mouse CD45. https://www.biolegend.com/en-us/products/apc-anti-mouse-cd45-antibody-97
(17) PerCP anti-mouse F4/80 Antibody. https://www.biolegend.com/en-us/products/percp-anti-mouse-f4-80-antibody-4302
(18) PE anti-mouse CD206. https://www.biolegend.com/en-us/products/pe-cyanine7-anti-mouse-cd206-mmr-antibody-8631?GroupID=BLG9506
(19) PE anti-mouse/human CD44 Antibody. https://www.biolegend.com/en-us/products/pe-anti-mouse-human-cd44-antibody-2206
(20) APC/Cyanine7 anti-mouse CD62L Antibody. https://www.biolegend.com/en-us/products/apc-cyanine7-anti-mouse-cd62l-antibody-3934

Eukaryotic cell lines

Policy information about cell lines and Sex and Gender in Research

Cell line source(s)

Mouse 4T1 cells was originally obtained from ATCC.
Luc+ 4T1 cells were obtained from OBiO Technology (Shanghai) Corp.,Ltd.

Authentication	Identity of the cell lines were frequently checked by their morphological features.
Mycoplasma contamination	All cell lines were tested for mycoplasma contamination. No mycoplasma contamination was found
Commonly misidentified lines (See ICLAC register)	No commonly misidentified cell lines were used.

Animals and other research organisms

Policy information about studies involving animals; ARRIVE guidelines recommended for reporting animal research, and Sex and Gender in Research

Laboratory animals	Female BALB/c mice (4-6 weeks) were purchased from Shanghai Sakas Biotechnology Co., Ltd. All mice were housed in SPF-grade pathogen-free facilities, 12 light/dark cycle, 40%- 70% relative humidity, temperature ~25°, with free access to standard food and water.
Wild animals	The study did not involve wild animals.
Reporting on sex	In this study, female mice were employed to construct the tumor model because the breast cancer mainly occurred in females.
Field-collected samples	The study did not involve samples collected from field.
Ethics oversight	All animal experiments were under the context of the animal protocols approved by the Institutional Animal Care and Use Committee guidelines in Shanghai Tenth Peoples' Hospital. All mice were kept in accordance with the e policies on animal research of the National Ministry of Health.

Note that full information on the approval of the study protocol must also be provided in the manuscript.

Flow Cytometry

Plots

Confirm that:

- [x] The axis labels state the marker and fluorochrome used (e.g. CD4-FITC).
- [x] The axis scales are clearly visible. Include numbers along axes only for bottom left plot of group (a 'group' is an analysis of identical markers).
- [x] All plots are contour plots with outliers or pseudocolor plots.
- [x] A numerical value for number of cells or percentage (with statistics) is provided.

Methodology

Sample preparation	For all samples, cells were first stained with antibodies against surface antigens. In some experiments, cells were subsequently fixed, permeablized andstained for intracellular antigens. For tissue sample, the tissue was first mechanically disrupted from mice and divided into small pieces and homogenized in cold staining buffer to form single cell suspensions in the presence of digestive enzyme.
Instrument	BD Fortessa X20
Software	FlowJo software package (version 10.5.2, BD Life Sciences, Ashland, Oregon, USA).
Cell population abundance	No sorting was performed.
Gating strategy	Generally, cells were first gated on FSC/SSC. Singlet cells were gated using FSC-H and FSC-A. Surface antigen gating was performed on the live cell population.

- [x] Tick this box to confirm that a figure exemplifying the gating strategy is provided in the Supplementary Information.
